# Patients’ satisfaction with HIV and AIDS care in Anambra State, Nigeria

**DOI:** 10.1371/journal.pone.0206499

**Published:** 2018-10-26

**Authors:** Chukwuma David Umeokonkwo, Patricia Nonye Aniebue, Chima Ariel Onoka, Adaoha Pearl Agu, Muawiyyah Babale Sufiyan, Lawrence Ogbonnaya

**Affiliations:** 1 Department of Community Medicine, Federal Teaching Hospital Abakaliki, Ebonyi State, Nigeria; 2 Nigeria Field Epidemiology Training Program, Abuja, Nigeria; 3 Department of Community Medicine, University of Nigeria Enugu Campus, Enugu, Enugu State, Nigeria; 4 Department of Community Medicine, Ebonyi State University, Abakaliki Ebonyi State, Nigeria; 5 Department of Community Medicine, Ahmadu Bello University Zaria, Kaduna State, Nigeria; Boston University School of Public Health, UNITED STATES

## Abstract

**Introduction:**

HIV and AIDS care requires frequent visits to the hospital. Patient satisfaction with care services during hospital visits is important in considering quality and outcome of care. Increasing number of patients needing treatment led to the decentralization of care to lower level hospitals without documented patient perception on the quality of services. The study determined and compared patient satisfaction with HIV and AIDS care services in public and private hospitals and identified the factors that influence it.

**Method:**

This was a cross-sectional comparative study of patients receiving antiretroviral treatment in public and private hospitals in Anambra State. The sampling frame for the hospitals consisted of all registered public and private hospitals that have rendered antiretroviral services for at least one year. There were three public urban, nine public rural, eleven private urban and ten private rural hospitals that met the criteria. One hospital was selected by simple random sampling (balloting) from each group. Out of a total of 6334 eligible patients (had received ART for at least 12 months), 1270 were recruited by simple random sampling from the hospitals proportionate to size of patient in each hospital. Adapted, validated and pretested Patient Satisfaction Questionnaire (PSQ18) was interviewer-administered on consenting patients as an exit interview. A Chi-square test and logistic regression analysis were conducted at 5% level of significance.

**Result:**

There were 635 participants each in public and private hospitals. Of the 408 patients who had primary education or less, 265(65.0%) accessed care in public hospitals compared to 143(35.0%) who accessed care in private hospital (p<0.001). Similarly, of the 851 patients who were currently married, 371 (43.6%) accessed their care in public compared to 480 (56.4%) who accessed care in private (p<0.001). The proportion of participants who were satisfied were more in public hospitals (71.5%) compared to private hospitals (41.4%). The difference in proportion was statistically significant (χ^2^ = 116.85, p <0.001). Good retention in care [AOR: 2.3, 95%CI: 1.5–3.5] was the only predictor of satisfaction in public hospitals while primary education [adjusted odds ratio (AOR); 2.3, 95%CI: 1.5–3.4], residing in rural area [AOR: 2.0, 95%CI: 1.4–2.9], and once-daily dosing [AOR: 3.2, 95%CI: 2.1–4.8] were independent predictors of patient' satisfaction among private hospital respondents.

**Conclusion:**

Satisfaction was higher among patients attending public hospitals. Patient’s satisfaction was strongly associated with retention in care among patients in public hospitals. However, in private hospitals, it was influenced by the patient’s level of education, place of residence, and antiretroviral medication dosing frequency.

## Introduction

HIV is the fifth leading cause of global Disability Adjusted Life Years (DALY) in 2010 [1,2]. An estimated 0.8% of adults aged 15–49 years worldwide are living with HIV, although the burden of the epidemic continues to vary considerably between countries and regions [3–5]. Sub-Saharan Africa remains most severely affected, with nearly 1 in every 20 adults (4.9%) living with HIV and accounting for 69% of the people living with HIV worldwide [3]. Nigeria had 3.6 million people living with HIV infection [6]. In Anambra State, the prevalence of HIV infection has more than doubled in the last few years. According to the Nigeria National HIV sentinel survey of 2010, the prevalence of the disease rose to 8.7% from the previous 3.5% in 2003, while the national prevalence declined from 5.3% to 4.1% within the same period [7].

Over 17 million people were receiving treatment globally as at the end of 2015 [8]. By 2015, in Nigeria, only 28% of those infected were accessing antiretroviral therapy[6]. In Anambra State, the number needing treatment has been on the rise [7].

HIV and AIDs has evolved over the years from an acute deadly disease to a chronic disease requiring regular clinic visits for medical consultation, laboratory testing and medication refills [9–11]. The patient needs to be self-motivated and satisfied in order to remain committed to these activities. Patient satisfaction is a multidimensional construct that focuses on different aspects of health service delivery and outcome [12]. It is a potent tool for evaluating care services and validating the quality of care provided. Information obtained therein helps health administrators to identify areas of improvement such as patient education, health worker-patient relationship, program planning, follow up and clinic organization in order to rapidly improve the quality of health service delivery and its expected outcome [13]. Patient’s satisfaction can be used as an indicator of health care quality because the more satisfied patient is the more likely patient to cooperate with the health care provider and have a higher level of continuity with the provider which in turn improves clinical outcome [14,15].

It has been argued that patients’ satisfaction rating is both a measure of care and a measure of the person that provided the rating [16]. Patient satisfaction rating can measure the different aspects of the medical services received or different specific dimensions of the satisfaction or the overall level of satisfaction of total package often referred to as global satisfaction. There are eight dimensions of patient satisfaction frequently reported in most satisfaction surveys and these dimensions include; interpersonal manner, technical quality, accessibility/convenience, finance, efficacy/outcome, continuity, physical environment and availability[16].

Certain patient characteristics are known to correlate with the global patient satisfaction rating. Older age patients were significantly more satisfied than younger patients [17–20]. This, however, is not completely linear, as it has been found that the global patients’ satisfaction rating start declining from the age of 65–80 years of life [18]. Healthier patients and those with less education were significantly more satisfied than patients with poorer health status or more education [17,20]. Living in the rural or urban area was significantly associated with younger patients but not with older patients [17]. However patient characteristics like gender, living alone or with others, or whether or not the questionnaire was self-administered or interviewer-administered were not known to be associated with patients’ satisfaction [17,19].

There are many studies on patient satisfaction with health care services in general and HIV services in particular. Most studies were carried out on patients receiving treatment from one hospital [13,21–24]. A study in Tanzania compared patient satisfaction with HIV related laboratory services in public and private laboratories while another study in north-central Nigeria compared patient satisfaction among public and private secondary level hospital and found no difference [25,26]. This study was conducted to determine and compare the level of satisfaction with HIV and AIDS care services among participants accessing care in public and private hospitals and to identify the factors that influenced their satisfaction.

## Methods

### Study sites

**Fig 1:**
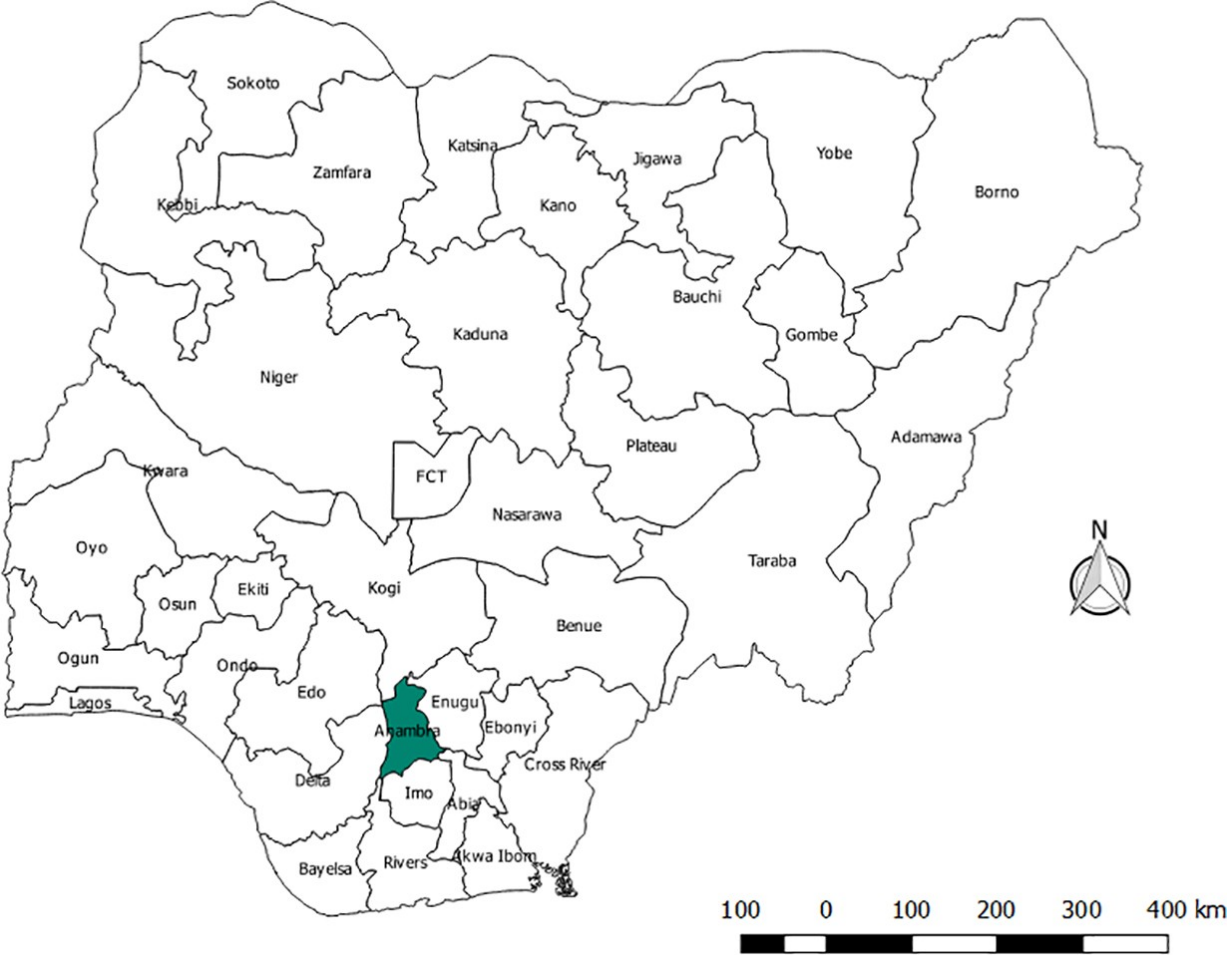
Map of Nigeria showing Anambra State, the site of the study.

The study took place in Anambra State, southeast Nigeria (Fig 1). Anambra State was the only State in the South East that had a prevalence of over 8% increase from the prevalence of 5.6% in 2008 to 8.7% in 2010 and one of the five states in the country that had prevalence of over 8% (Akwa Ibom 10.9%, Bayelsa 9.1%, Benue 12.7% and FCT 8.6%) [7]. Anambra is one of the most densely populated states and among the most urbanized areas in the country [27]. The urban/rural comparison of HIV prevalence rate in Anambra shows a wide variation of 10.1% urban against 4.7% rural prevalence. The state prevalence has consistently remained above the median national prevalence since 2008 [7].

The hospital system in Nigeria is categorized based on the complexity of the services they provide into primary, secondary and tertiary hospitals. The primary level hospitals are the first level of contact of the population to the health system. They provide preventive, health promotion services and less complex curative services. They usually manned by a medical doctor where available but mostly by nurses and community health officers in most places. The secondary level hospitals are the second level of care. More specialized care is provided here. They have laboratory support, surgery, and other specialist services. Cases needing more than primary care are referred to these hospitals. They often cover wider catchment areas like Local Government Areas and Districts. Tertiary hospitals provide the highest level of specialized care in the health system. They include the teaching and specialist hospitals. In addition to care, they also exist to carry out research and training of doctors and other health workers.

The public hospitals are funded and managed by Government primarily to provide health services to the populace. Whereas, the private hospitals are funded by individuals and organizations to provide health services as well as make a profit. In Nigeria, the treatment of HIV started with few tertiary hospitals but later cascaded down to public secondary hospitals and then to private secondary hospitals. These hospitals were all supported by partners to provide free HIV treatment. HIV drugs have remained free in both public and private hospitals. However, following the withdrawal of funding for some laboratory tests, patients have to pay for these services in both public and private facilities. The amount paid for laboratory services was lower public hospitals due to government subsidies than in private hospitals.

There were 51 secondary level hospitals in Anambra State that provide comprehensive HIV services. Eighteen hospitals were just recently activated about the time for data collection and had provided HIV services for less than one year and were excluded. The remaining 33 were stratified into publicly and privately owned. In each group, they were also stratified based on the location of practice–urban and rural. There were three public urban, nine public rural, eleven private urban and ten private rural hospitals. In each of these strata, one hospital is selected by balloting to participate in the study. In all two public and two private hospitals were selected for the study.

These were two publicly-owned hospitals (General Hospital Onitsha and General hospital Ekwulobia) and two privately-owned, faith-based hospitals (St Joseph’s Hospital Adazi and St Charles Borromeo Hospital Onitsha). These hospitals received technical support from Non-Governmental Organizations.

General Hospital Onitsha commenced comprehensive ART care and treatment in 2007, runs HIV Clinics twice weekly and had 2438 patients on antiretroviral treatment at the time of the study. St Charles Borromeo Specialist Hospital Onitsha commenced HIV comprehensive care and treatment in July 2005, runs daily HIV clinics and had a total of 2482 patients receiving antiretroviral treatment for HIV infection. General Hospital Ekwulobia commenced provision of comprehensive HIV services in March 2007, runs the HIV clinic twice weekly and had 509 patients currently on antiretroviral treatment. St Joseph’s Hospital Adazi commenced HIV comprehensive care and treatment services in 2007, runs an integrated HIV clinic twice weekly, and had 905 patients on antiretroviral treatment.

### Study design and sampling

The cross-sectional comparative study was conducted between April and August 2015 among adult HIV positive outpatients who had taken antiretroviral treatment for at least one year. The sample size was estimated using the formula for two sample proportion,[28] a power of 80%, the minimum effect size of 3.4%, 95% confidence level and proportion of satisfaction among public hospital participants of 67.5% as reported in a previous study,[25] and a 15% non-response rate. A total of 635 participants were recruited per group.

$$\begin{array}{c} n=[{Z\alpha \sqrt{2P1(1-P1})-Z(1-\beta )\sqrt{P1(1-P1)+P2(1-P2)}]}^{2}\\ {\left(P1-P2\right)}^{2} \end{array}$$

A list of secondary level hospitals offering comprehensive HIV treatment services in Anambra State was obtained and four hospitals were recruited to participate in the study as described above. The list of all patients who are currently on ART (antiretroviral treatment) in each hospital was generated using the facility-based National ART Register. The patient unique ART numbers were captured into a Microsoft Excel workbook for the hospital and ordered in ascending order. This formed the sampling frame for this stage of sampling. The sample size for each group–public and private, were proportionally allocated to the facilities based on the number of patients that were currently receiving ART. These were: General Hospital Onitsha (2438), General Hospital Ekwulobia (509), St Charles Borromeo Hospital Onitsha (2482) and St Joseph’s Hospital Adazi (905). The proportionate allocation of the sample size for each hospital (n_h1_) was determined using the formula:

$$n_{h1}=\left(\frac{x_{h1}}{x_{ht}}\right)\times n_{st}$$

Where *x*_*h1*_ = number of eligible patients in an index hospital, *x*_*ht*_ = total number of eligible patients in the hospital group (e.g. private hospitals), and *n*_*st*_ = calculated sample size for the hospital group. Hence, the sample sizes used for the study were 525, 110, 465 and 170 for GHO, GHE, SCBHO, and SJHA, respectively.

A table of random numbers was used to select the sample for each hospital. The first number was selected by dropping a pencil at the center of the table of random numbers and moving down the columns from the top down thereafter. Groups of four digits were used. The pool selected was subsequently matched against the appointment list on each clinic day. Those that attended clinic were approached by trained research assistants after consultation with their clinicians. The study was introduced to them, written informed consent was obtained and the interview was conducted in a room with audio-visual privacy. Before administering the questionnaire, the participants were asked if they had been previously approached for the same study in the last three months. In addition, information about their previous visits, regimen type, and dosing frequency, was extracted from participants’ case notes. The numbers of those interviewed and those that declined participation were struck out from the list. After the first two months, those that did not attend the clinic were replaced using the simple random sampling with a table of random numbers described above.

### Study instrument

Marshall and Hays’ Patient Satisfaction Questionnaire short form (PSQ 18)[29]was adapted to assess satisfaction and it was pretested among adult HIV positive outpatient receiving treatment in Ebonyi State (a state in the same geographic region, but situated about 180km away from the study sites). The pretesting led to further modification of the questionnaire before use. The reliability of the tool used to assess satisfaction was tested with Cronbach alpha and the result was 0.78. The questions were on a 5-point Likert scale; each domain has positively and negatively structured questions, with a minimum of two and a maximum of four questions. The seven domains of satisfaction were assessed together with patient socio-demographic and clinical information. The study instrument also assessed adherence to antiretroviral treatment using patient self-report. Retention in care was estimated using a 3-month visit constancy method [30–32].

### Data analysis

Epi Info version 7.0 was used for the data analysis. The responses to the questions in each domain were scored, aggregated and categorized as described by developers of the tool [29]. Participants that scored at least 80% of the maximum expected scores for each domain were classified as satisfied while those with scores less than 80% were classified as dissatisfied.

The participants were asked how many doses they had missed in the preceding four-week period. The dosing frequency and the reported number of missed doses were used to estimate the rate of adherence to antiretroviral treatment (ART) calculated as a percentage using the formula[33–35] below:

$$Adherence\;to\;treatment\;(\%)=\;\frac{Total\;number\;of\;doses\;of\;ART\;taken\;}{Total\;number\;of\;prescribed\;doses\;of\;ART}\times \frac{100}{1}$$

where

Totalprescribeddose=dosingfrequency(howmanydosesperday)X28days(4weeks)

TotalnumberofdosesofARTtaken=TotalnumberofprescribeddosesofART–totalnumberofmissedARTdosesinthelast28days(4weeks)

Those that had adherence greater than or equal to 95% were classified as good adherence, otherwise, they were classified as poor adherence.

Three months visit constancy method was used because the appointment scheduling team in the study area schedules refill appointments every two months. Three months method is more sensitive compared to a four-month method which is usually used in a context where refill visits are scheduled every three months. The 3-month visit constancy method counts the number of the 3-month interval with at least one ‘kept clinic visit’ during a measurement period. The measurement period was one year prior to the study time. A ‘kept clinic visit’ was defined as a scheduled visit in which the patient attended, met with and received antiretroviral (ARV) drugs prescription from a health worker who is qualified to prescribe ARV drugs to the patients. The information was extracted from the participants’ record to avoid recall bias and ensure accuracy. The participants were scored 1, 2, 3, or 4 depending on the number of quarters with at least one kept visit. A participant that had at least one kept visit each in three quarters scored 3 out of a total of 4 possible scores for example. For the purpose of further analysis, the scores were categorized into ‘adequate retention’ (those that scored 4) and ‘inadequate retention’ (those that scored less than 4).

The proportions were compared across public and private hospitals using chi-square test. We assessed the relationship between overall satisfaction and sociodemographic/clinical characteristics using Chi-square test. The factors that were associated with overall satisfaction in bivariate analysis were examined with a multiple logistic regression model at 5% level of significance.

### Ethical consideration

Ethical approval number 12/02/2015-23/02/2015 dated 23^rd^ February 2015 was obtained from the Research and Ethics Committee (REC) of Federal Teaching Hospital Abakaliki, (FETHA) Ebonyi State Nigeria. Permission was also obtained from the Anambra State Ministry of Health through the Health Management Board and from the managers of the private hospitals. We obtained written informed consent from all the participants. The exit interviews were conducted in rooms with audio-visual privacy. The participants’ data were anonymized and handled with utmost confidentiality throughout the study.

## Results

The mean age of the participants was 40.1years (± 9.9years) with females constituting 71.7%, 68.7% were urban dwellers, 88.3% were currently employed and only 14.4% had attained post-secondary education. Participants that accessed care in public hospitals were comparable in their sociodemographic characteristics to those that accessed care in private hospitals, except with regards to their marital status and educational level. (Table 1)

**Table 1:** Socio-demographic characteristics of the patients accessing HIV treatment services in Anambra State Nigeria, 2015.

Variable	Public Health Facility (n = 635) Frequency (%)	Private Health Facility (n = 635) Frequency (%)	Chi square (χ^2^)	p-value
**Age (years):**				
< 30	83 (13.1)	79 (12.4)		0.913*
30–39	234 (36.9)	246 (38.7)	0.53	
40–49	198 (31.2)	191 (30.1)		
≥ 50	120 (18.9)	119 (18.7)		
**Mean age ± SD (years)**	40.14 ± 9.84	40.11 ± 9.92		0.960^#^
**Gender**				
Male	170 (26.8)	189 (29.8)	1.40	0.236*
Female	465 (73.2)	446 (70.2)		
**Place of Residence**				
Rural	202 (31.8)	195 (30.7)	0.18	0.672*
Urban	433 (68.2)	440 (69.3)		
**Educational level:**				
No formal education	24(3.7)	12(1.9)		<0.001*
Primary education	241(38.0)	131(20.6)	53.96	
Secondary education	289(45.5)	390(61.4)		
Post-Secondary education	81(12.8)	102(16.1)		
**Marital status:**				
Currently Married	371 (58.4)	480 (75.6)		<0.001*
Single	125 (19.7)	96 (15.1)	53.47	
Widowed	127 (20.0)	48 (7.6)		
Divorced	12 (1.9)	11 (1.7)		
**Employment Status:**				
Currently Employed	550 (86.6)	572 (90.1)	3.70	0.054*
Currently Unemployed	85 (13.4)	63 (9.9)		
**Religion**				
Christianity	630 (99.2)	633 (99.7)		0.512*
Islam	3 (0.5)	1 (0.2)	1.34	
African Traditional Religion	2 (0.3)	1 (0.2)		

*p-value of Chi-square

^#^p-value of t test

SD = standard deviation

Patients in the public health facilities reported better satisfaction in all the seven domains of satisfaction assessed [Table 2]. On the general satisfaction domain, public hospital patients were more satisfied (71.5%) than those in private hospitals (41.4%) and the difference between them was statistically significant (p <0.001). In both hospital types, however, less than 50% of the participants were satisfied with the technical quality of their health care providers [public (49.7%), private (35.4%)] and time spent with the doctor [public (39.1%), private (33.4%)]. A higher level of satisfaction in the manner of approach of the health care providers and effective communication in both hospital types. The widest variation was observed in the cost of services received: more participants from the public hospitals were satisfied (76.7%) compared to those from private health facilities (34.8%) and this difference was statistically significant (p <0.001). [Table 2]

**Table 2:** Patients’ satisfaction with HIV treatment services received in public and private hospitals in Anambra State Nigeria, 2015.

Satisfaction Domains	Public Health Facility (N = 635) Frequency (%)	Private Health Facility (N = 635) Frequency (%)	p-value*
General satisfaction	454 (71.5)	263 (41.4)	<0.001
Technical quality	316 (49.7)	224 (35.3)	<0.001
Time spent with doctor	248 (39.1)	212 (33.4)	0.036
Manner of approach	555 (87.4)	476 (75.0)	<0.001
Effective communication	612 (96.4)	556 (87.6)	<0.001
Cost of services received	487 (76.7)	221 (34.8)	<0.001
Accessibility and convenience	392 (61.7)	314 (49.4)	<0.001

*p-value based on chi-square test

Table 3 relates satisfaction to socio-demographic/clinical characteristics of participants in public and private hospitals. To understand how these variables interact with each other, a logistic regression model was used to examine variables that interacted with satisfaction at 10% level of significance [Table 4]. Based on the logistic regression model, only retention in care remained significantly associated with patient’s satisfaction (p<0.001). Participants who had good retention in public hospitals were twice more likely to be satisfied with services received from the hospitals compared to those who had poor retention.

Unlike in the public hospitals, the level of education, place of residence and ART medication dosing frequency were significantly associated with patient’s satisfaction in private hospitals [Table 4]. Participants that had primary education or less had higher odds (AOR:2.3, 95%CI: 1.51–2.86) of being satisfied with services received compared those that had secondary education or higher among the private health facilities. Additionally, participants living in rural area had higher odds (AOR:2.0, 95%CI: 1.37–2.86) of being satisfied compared to those that were living in urban areas and those participants who took their medication once daily had higher odds (AOR:3.2, 95%CI: 2.11–4.85) of being satisfied compared to those that took their medications twice in a day.

**Table 3:** Relationship between patient’s satisfaction and socio-demographic /clinical characteristics among respondents in public and private hospitals in Anambra State Nigeria, 2015.

Variable	Public hospital n = 635			Private hospital n = 635		
	Satisfied (%)	χ^2^	p-value	Satisfied (%)	χ^2^	p-value
**Age**						
Less than 35yrs	135 (66.8)	3.16	0.075	80(40.2)	0.18	0.674
35yrs and older	319 (73.7)			183(42.0)		
**Gender**						
Male	119 (70.0)	0.26	0.614	77(40.7)	0.05	0.822
Female	334 (72.0)			186(41.7)		
**Marital status**						
Currently married	270 (72.8)	0.72	0.397	187(39.0)	4.90	0.027
Not currently married	184 (69.7)			76(49.0)		
**Employment status**						
Employed	398 (72.4)	1.52	0.218	238(41.6)	0.09	0.768
Unemployed	56 (65.9)			25(39.7)		
**Place of residence**						
Rural	147 (72.8)	0.24	0.027	111(56.9)	27.89	<0.001
Urban	307 (70.9)			152(34.5)		
**Education**						
Primary education or less	195 (73.6)	0.97	0.324	85(59.4)	24.71	<0.001
Secondary education and more	259 (70.0)			178(36.2)		
**Medication dosing frequency**						
12hrly	327 (71.8)	0.04	0.848	171(34.4)	46.33	<0.001
24hrly	127 (70.9)			92(62.7)		
**Regimen type**						
First line	448 (72.0)	4.18	0.041	252(41.2)	0.40	0.525
Second line	6 (46.2)			11(47.8)		
**Experienced stock out**						
Yes	216 (71.8)	0.02	0.888	9(33.3)	0.76	0.383
No	238 (71.3)			254(41.8)		
**Disclosure status**						
Yes	408 (72.1)	0.89	0.347	235(41.9)	0.44	0.506
No	46 (66.7)			28(37.8)		
**Transportation cost (N)**						
Less than 1000	424 (71.6)	0.07	0.795	232(41.1)	0.17	0.684
More than 1000	30 (69.8)			31(43.7)		
**Retention**						
Good Retention	384 (75.3)	18.34	<0.001	210(40.8)	0.46	0.497
Poor Retention	70 (56.0)			53(44.2)		
**Adherence to treatment**						
Good Adherence	418 (72.6)	3.51	0.061	251(41.5)	0.03	0.872
Poor Adherence	36 (61.0)			12(40.0)		

**Table 4:** Multiple logistic regression of factors associated with patients’ satisfaction in public and private hospitals in Anambra State Nigeria, 2015.

Independent variables	Public hospital			Private hospital		
	Adjusted Odds Ratio	95% CI	p-value	Adjusted Odds Ratio	95% CI	p-value
**Age**						
Less than 35years	0.793	0.546–1.151	0.222			
35 years and older	1					
**Education**						
Primary education or less				2.254	1.505–3.374	<0.001
Secondary education or more				1		
**Place of residence**						
Rural	1.157	0.789–1.697	0.455	1.981	1.373–2.856	<0.001
Urban	1			1		
**Marital status**						
Currently married				0.723	0.489–1.070	0.105
Not currently married				1		
**Regimen type**						
First line	2.467	0.795–7.654	0.118			
Second line	1					
**Medication dosing frequency**						
24 hourly				3.200	2.112–4.847	<0.001
12 hourly				1		
**Adherence**						
Good adherence	1.580	0.893–2.796	0.116			
Poor adherence	1					
**Retention in care**						
Good retention	2.320	1.535–3.505	<0.001			
Poor retention	1					

## Discussion

This study set out to examine the difference in satisfaction with services among patients receiving care in public and private hospitals across various domains namely, general satisfaction, technical quality, time spent with the doctor, the manner of approach, effective communication, cost of services received and accessibility and convenience. Overall, the analysis shows a higher level of patients’ satisfaction with services across all domains examined among study participants receiving care in public hospitals, and the influence of various socio-demographic and clinical characteristics on satisfaction.

Generally, the high level of satisfaction with services observed among the participants from the public hospitals compares with earlier studies focused on public hospitals reported in Nigeria[23,25] and elsewhere in Cameroon and Zambia [24,36] Comparatively, the difference in the level of satisfaction reported by participants is significant and contrasts with findings from earlier studies in Nigeria [25,37]. Two major attributes of these hospitals relate to the findings, namely, the availability of public subsidies and the characteristics of the users.

Regarding subsidies, it has been argued that where services are largely subsidized by the government, users generally report a high level of satisfaction due to their little expectation from the system, covering various domains examined. Users are also likely to accept levels of services they may not have accepted if the services were fully paid for as obtains in private hospitals. While the influence of cost on satisfaction was generally noted as negative, the relationship was stronger for private hospitals that have no subsidies for laboratory services. The effect of the cost of services received on the level of satisfaction has been reported [22]. While drugs remain free or hugely subsidized, the implications of the cost of laboratory services is that unsubsidized care does not only affect satisfaction but has the potential to drive households into poverty.

Regarding the characteristics of users, individuals with higher educational status were less likely to use public hospitals and were also less likely to be satisfied. It is also known that people that are more able to pay, those with better education and living standards, prefer the more sheltered private hospitals. Satisfaction has been opined as the difference between expectation and experience, which on its own is affected by users’ socio-demographic characteristics. The higher education one gets, the more one expects from a system. In this population, individuals with higher education (secondary school and higher) were more likely to access their services from private hospitals. This finding is keeping with that earlier reported [17,19,20] but differs with other findings [17,18]. Issues such as place of residence (urban and rural) were also significant determinants of satisfaction for participants in the private hospitals.

There were also similarities in patients’ satisfaction in specific domains that warrant attention. The proportions that were satisfied were less than 50% in both public and private hospitals in technical quality and time spent with the doctor domains. This could mean the patients’ perception of the technical competency of their clinicians were suboptimal. It is also an indicator that most of the patients were not satisfied with the amount of time they spent interacting with their clinicians in both hospital types. These aspects warrant further research exploration to understand its relationship with attitudes of caregivers, motivation, as well as adequacy and workload.

Finally, this study also showed that participants with better satisfaction in the public hospitals had better retention attributes. This finding supports earlier studies that established the relationship between retention in HIV care and patient satisfaction [21,38]. Also, retention has been previously documented as less of a problem in private hospitals than in public hospitals in the same region of Nigeria [39]. Ensuring retention is very important in HIV programming because retention in care is a critical quality indicator in HIV management. In the private hospitals, dosing frequency was more of a problem with regards to patient experiences. The findings suggest the need to transition patients who can take drugs with fewer dosing schedules to such regimen.

## Conclusion

Patients’ satisfaction was significantly higher among the participants accessing HIV care in public hospitalsin Anambra State compared with their counterpart in the private hospitals. Retention in care was the only factor significantly associated with patients’ satisfaction among the public health facilities’ participants, while place of residence, education, and HIV medication dosing frequency were predictors of patients’ satisfaction among the private hospitals. Cost remains an important determinant of satisfaction and given the chronic need for treatment, subsidies are still necessary for patients in private hospitals. Programme managers in public hospitals should examine and ensure recognized interventions that influence retention, given the relationship with satisfaction, in order to bring this important quality indicator to an optimum level. Positive experiences promoting satisfaction in both public and private hospital should be shared across various facility types to optimize the quality of life of all persons living with HIV and AIDS.

## Supporting information

S1 File Questionnaire. (PDF)
