# Supplementary material for: Patients’ satisfaction with HIV and AIDS care in Anambra State, Nigeria
Source: PLoS One. 2018 Oct 26;13(10):e0206499. doi: 10.1371/journal.pone.0206499 (PMC6203402; doi:10.1371/journal.pone.0206499)
Supplement: S1 File — (PDF) [file pone.0206499.s001.pdf]

Retention in care, Adherence to Treatment / Patients' Satisfaction among HIV Patients: Comparison of Public and Private Health Facilities in Anambra State

SECTION 1: SOCIO DEMOGRAPHIC CHARACTERISTICS

Facility Type…………

Hospital ………….

1. In the last one year have you been hospitalized for more than one month? No [ ] Yes [ ] If yes stop the interview
2. Sex Male [ ] Female [ ]
3. Age as at the last birthday ------------
4. Marital Status: Currently Married [ ] Single [ ] Divorced [ ] Widow/widower [ ] Separated [ ]
5. Religion: Christianity [ ] Muslim [ ] African Traditional Religion [ ] Others Specify…….
6. Employment Status: Trader [ ] Unemployed [ ] farmer [ ] Civil Servant [ ] Self-employed [ ] Others Please specify ……………..
7. Where do you leave presently? Urban area [ ] Rural Area [ ]
8. What is the highest level of Education you have completed? No education [ ] Primary Education [ ] Secondary Education [ ] Tertiary Education [ ] Postgraduate Education [ ]

SECTION 2: CLINICAL CHARACTERISTICS

9. How long have you been diagnosed HIV positive ------------(months)
10. How long ago have you been receiving care and treatment for HIV (before) you started ART on lifelong basis? ……………..(in months)
11. How many years have you been on antiretroviral treatment? 1yr [ ] 2yrs [ ] 3yrs [ ] 4yrs [ ] 5yrs [ ] >5yrs [ ] (Please Specify)………
12. How would you rate your state of health today? Excellent [ ] Very Good [ ] Good [ ] Bad [ ] Very Bad [ ] Worst [ ]
13. How much time did you spend waiting to see your doctor today? Less than 30mins [ ] 30mins – 1hr [ ] 1-2hrs [ ] 2-3hrs [ ] 3-4hrs [ ] >4hrs [ ] (Please specify……….)

SECTION 3: PATIENT SATISFACTION

Instructions: After reading each question, circle the answer that best describes your opinion. The order of the answers varies between the questions, so take a moment to read each question carefully. I know that sometimes answers may not describe how you feel exactly, so please pick the answer that most closely describes your opinion.

		Strongly Satisfied	Satisfied	Neither Satisfied nor Dissatisfied	Dissatisfied	Strongly Dissatisfied
14	How satisfied are you with the services you received in this hospital today?					
15	How satisfied are you with the amount of time you spent waiting to see your doctor in this hospital?					
16	How satisfied are you with the responsiveness of health care workers to your questions and requests?					
17	How satisfied are you with the medical confidentiality and respect of your privacy in this hospital?					
18	How satisfied are you with the consultation, explanation, and guidance you have received from doctors in this hospital?					
19	How satisfied are you with the consultation, explanation, and guidance you have received from nurses in this hospital?					
20	How satisfied are you with the consultation, explanation, and guidance you have received from pharmacists workers in this hospital?					
21	How satisfied are you with the consultation, explanation, and guidance you have received from laboratory scientists in this hospital?					
22	How would you rate the convenience in using medical services, such as laboratory tests services?					
23	How would you rate the convenience in using medical services, such as adherence counselling services					
24	How would you rate the convenience in using medical services, such as pharmaceutical services					
25	How would you rate the competency of health care workers					
26	How satisfied were you with the choices you had in decisions affecting your health care?					

		Strongly Agree	Agree	Neither Agree Nor Disagree	Disagree	Strongly Disagree
27	The time you had with the [doctor/other health professional] was too short					
28	I have to pay more for my medical care than I can afford					
29	I find it hard to get an appointment for medical care right away.					
30	The medical care I have been receiving is just about perfect					
31	My doctors treat me in a very friendly and courteous manner					
32	Those who provide my medical care sometimes hurry too much when they treat me					
33	I am able to get medical care whenever I need it.					
34	I would recommend this hospital to my friends or family who need this type of service I received.					
35	Doctors are good about explaining the reason for medical tests					
36	I think my Doctor's office has everything needed to provide complete medical care					
37	I feel confident that I can get medical care I need without being set back financially					
38	Sometimes doctors make me wonder if their diagnosis is correct					
39	Doctors act too business-like and impersonal toward me					
40	Where I get medical care, people have to wait too long for emergency treatment					
41	I have some doubts about the ability of the doctors who treat me					
42	Doctors usually spend plenty of time with me					
43	I am dissatisfied with some things about the medical care I receive					
44	When I go for medical care, they are careful to check everything when treating and examining me					
45	I have easy access to the medical specialists I need					

SECTION 4: ADHERENCE TO TREATMENT

46. How many times do you take your HIV medicine in a day? Twice (morning and evening) [ ] Once (only at Night) [ ] Others [ ] (please specify)……….

47. In the past four (4) weeks, how would you rate your ability to take all your HIV medicines as your doctor prescribed? Very poor [ ] Poor [ ] Fair [ ] Good [ ] Very Good [ ] Excellent [ ]
48. In the last four weeks, how many doses were you unable to take? None [ ] 1 dose [ ] 2 doses [ ] 3 doses [ ] 4 doses [ ] 5 doses [ ] >5doses [ ] (Please specify)…………..
49. Why were you unable to take those doses?{tick all that apply} Forgot [ ] Ran out of drugs [ ] Drug side effect [ ] Away from home [ ] could not hide [ ] difficulty taking pills at specified time [ ] confusion about how to take pills [ ] too many drugs to take [ ] Fasting [ ] Others [ ] (please specify)…………
50. How many pills do you take in a day (total of all the drugs including HIV medicine) …………..
51. What type of regimen are you taking? {Extract from patient folder} First line [ ] Second line regimen [ ] Salvage regimen [ ]
52. The percentage adherence based on recalled missed doses and dosing frequency.{to be calculated by the research assistant} Adequate (more than or equal to 95%) [ ] Inadequate (less than 95%) [ ]
53. Are there instances when you come to this hospital and you are not able to receive your antiretroviral drugs (HIV drugs)? Yes [ ] No [ ]
54. If Yes, how frequently in the last one year? once [ ] twice [ ] thrice [ ] more than 3X [ ]
55. Have you disclosed your HIV status to anyone? Yes [ ] No [ ]
56. If yes, who did you disclosed to? Friend [ ] Parents [ ] Sibling [ ] Spouse [ ] Colleague at work [ ] Neighbour [ ] Others [ ] Please specify……………..

SECTION 4: RETENTION IN CARE

57. Visit Constancy: Check the patient records for evidence of any prescription visit in each of these four quarters and indicate as appropriate.

1st Jan – 31st March 2014 [ ] 1st April to 30th June 2014 [ ]
1st July to 30th September 2014 [ ] 1st October to 31st December 2014 [ ]

Adequate Retention (100% visit in all quarters) [ ]
Inadequate (less than 100% visit in all the quarters) [ ]

58. How much do you spend on average to transport yourself from home to the hospital to access your drugs? Less than N100 [ ] N100 – N500 [ ] N600 – N1000 [ ] more than N1000 [ ] Please specify ………
